# Reviews

**Published:** 1886-04

**Authors:** 


### REVIEWS.

ANTHROPOID APES. By Robert Hartmann, Professor in the University of Berlin. Wiih illustrations. New York: D. Appleton & Co., 1886, being Vol. LII. of the "International Scientific Series."

This work may be regarded as a sort of review of our past and present knowledge of these interesting animals, whose proximity to the human species in anatomical structure and intelligence has been the theme of naturalists and physicians, and the bane of more orthodox people during the past twenty years. As such it presents a fair view of our existing knowledge on the subject, without demonstrating anything novel, or being, indeed, in any respect very exhaustive. The author has himself had the opportunity of dissecting all species of the anthropoid apes, and has made a laborious analysis of the muscular variations, but regarding the visceral, cranial and cerebral characters of these species, he adds little or nothing, and does not even give a full abstract of the results of others. The work is lacking in vigor and originality. It may lay claim to being a good compilation, and in some respects, at least, falls short of its models. Thus, the first chapter, the one dealing with the history of our knowledge of the subject, is undoubtedly a reproduction of the opening chapter of Huxley's "Man's Place in Nature," yet how vastly superior in elegance of diction and interest is the latter over Hartmann's lines.

It is possible—for we have not seen the original—that the defects we notice are due to the translation, which has struck one other reviewer * as presenting inherent evidences of clumsiness. We refer to this confirmation lest we might be deemed to be hypercritical. We have, in turning over the pages of of this book, come across a number of instances of bad translation, and one of these, at least, deserves to be immortalized. On page 197, the text reads *verbatim et literatum*; "Bishoff asserts that the frontal convolution is very slightly developed in the chimpanzee, orang and gibbon. 'Its great development in men,' Gewahrsmann writes, 'constitutes one of the most marked distinctions between the brains of apes and men." To this citation there is appended a reference to the work of Bischoff showing conclusively that the translator has created a new authority by the name of 'Gewahrsmann.' The fact is, that the reference in the original is to Bischoff, who is spoken of as the *authority* or *Gewahrsmann* for that statement. We know of but one other authority created by a pretender to foreign literary knowledge, and that was "Beitz," cited by Dr. Allan McLane Hamilton, and due to a similar misappreciation of a German abbreviation.

The illustrations, as a rule, are good, and a sufficiently large number accompany the text to illustrate the subject. But in one instance, at least,

---

* In the New York Times.

the author has shown a lack of judgment. In selecting a type of savage to test the approximation of the lower human forms to the higher apes, he pitches upon the Zulu king, Cetewayo, and two of his subjects. It would have been, in our opinion, bad enough to take any Zulu, which is one of the noblest types of the savage, but it was much worse to select one of the exceptionally ablest warriors and diplomatists that race has brought forth. And in representing the lowest human type, Hartmann has been equally unfortunate. We do not need to go five hundred yards from where we are now sitting to find a negro exhibiting a far more ape-like appearance than the "hairless Australian" of page 88. Under these circumstances, we are not surprised at finding Hartmann reluctant to admit the pithecoid resemblance of the lower human races, a resemblance that no physiognomist can fail to detect.

A number of the cuts seem to have been thrown in without any regard to the text, quite a number of non-anthropoid apes are thus represented. Whether this is a publisher's measure or not, we are unable to say ; if the fault of the author, it is certainly very bad taste, as it tends to give a preponderance to to the views of those who would widen the chasm between the anthropidæ and the anthropomorpha.

While we are not surprised to find the American authors who have written and in part, at least, made original observations on the cerebral and general anatomy of the chimpanzee and ourang-outang, not referred to, for it is rather the custom of our German friends to think of America somewhat as certain people in Judæa thought of Nazareth, yet we would venture to direct Professor Hartmann's attention to some exceedingly painstaking researches of Wilder, demonstrating that the brain of the anthropoid ape (chimpanzee) overlaps the cerebellum. In case he condescends to follow Wilder's methods or to accept his measurements, may, if he were but to see through an anthropoid skull in the sagittal plane, he will learn better than to offer his readers such a caricature as the woodcuts of a gorilla's brain in pages 193 and 196. Even a dog would blush to have so flat a basi encephalic axis as is there shown.

The very worst part of the book is that relating to the brain, and this is scarcely to be wondered at as the compiler, Bastian, is cited as the foremost investigator on the subject. The author says—on what authority, we are not informed—that in Mongolian, Indian and Negro race, the cerebellum is imperfectly covered by the cerebrum. If the human brain is treated in the same way as the afore-mentioned gorilla brain, such a result will be obtained, but not otherwise. Hartmann says that the gorilla has more complex convolutions than the chimpanzee, but the photograph does not compare over favorably with the brains of some of our chimpanzees. What he means by saying that "there are instances" where the fissure of sylvius is *not* overlapped by the speculum, we are at a loss to conjecture. In this connection we discover another example of the fearful translating ability of our "Gewahrsmann." "In these three anthropoids, as Bastian justly observes, it is less horizontal than in man, and occupies a position more like that which it takes in the black sea-cat monkey, the wanderers, and other macacas." (page 195).

On page 197 he has "a soft and thick anterior commissure of the third cerebral ventricle." On the next page is the following incomprehensible jargon: "In the lateral ventricles more of the characteristics described in the human brain are absent. The four eminences (?) resemble those of man, nor does the fourth cerebral ventricle present any remarkable differences of form." Aside from the nonsensical translation of "vierhügel" (corpora quadrigemina) into four eminences, which the context would lead one to fear, the translator regarded as contained in the lateral ventricles, these statements are wrong. The lateral ventricles are very similar, and the four less so in the anthropoid group and man.

We regard it as unfortunate that a prominent publishing house should publish so poor a translation. Of course the system followed in the international series, by which the translator remains anonymous is calculated to have a bad effect every way. In the first place, by not venturing to publish the name of the translator, the publishers make a *quasi* confession that they intrust their work to some mechanical drudge, ignorant of the first principles of the science with which the work to be translated deals. In the second place, this system engenders carelessness on the part of the translator, for as his name remains unknown, he has no reputation to lose or to gain, by a good or bad translation.

We think, taking it all in all, that the important subject with which Professor Hartmann's work deals admitted of a better and more fascinating dress, and demands a more careful presentation than that of the translation before us.

***

Essentials of Vaccination. By Dr. W. A. Hardaway. Published by J. H. Chambers & Co.—St. Louis. 1886.

This little volume is a compilation of the main facts relating to a very important subject, about which much has been written, *pro* and *con*, and clearly has a *raison d'etre* at the present moment.

When a scientist like Herbert Spencer is found among those arrayed against such a long-established fact as the protective efficacy of vaccination, we appreciate the necessity of taking up arms in its defence. This Dr. Hardaway has done, and in dedicating it to Dr. Frank P. Foster, pays a just tribute to the scientific services of the most distinguished vaccinographer of the day.

